# Effects of a Postural Exercise Program on Vertical Jump Height in Young Female Volleyball Players with Knee Valgus

**DOI:** 10.3390/ijerph19073953

**Published:** 2022-03-26

**Authors:** Valerio Giustino, Giuseppe Messina, Antonino Patti, Elvira Padua, Daniele Zangla, Patrik Drid, Giuseppe Battaglia, Antonio Palma, Antonino Bianco

**Affiliations:** 1 Sport and Exercise Sciences Research Unit, Department of Psychology, Educational Science and Human Movement, University of Palermo, 90144 Palermo, Italy; valerio.giustino@unipa.it (V.G.); giuseppe.messina17@unipa.it (G.M.); daniele.zangla@unipa.it (D.Z.); giuseppe.battaglia@unipa.it (G.B.); antonio.palma@unipa.it (A.P.); antonino.bianco@unipa.it (A.B.); 2 Department of Human Sciences and Promotion of Quality of Life, San Raffaele Roma Open University, 00166 Rome, Italy; elvira.padua@uniroma5.it; 3 Faculty of Sport and Physical Education, University of Novi Sad, 21000 Novi Sad, Serbia; patrikdrid@gmail.com

**Keywords:** vertical jump height, vertical jump performance, biomechanics, sport performance, postural exercises, volleyball, body posture, knee valgus

## Abstract

Background: Although a knee valgus position is related to the increase in injury risk in volleyball players, there is a lack of studies on the relationship between knee valgus and vertical jump (VJ) performance. Hence, the aim of this study was to investigate the effects of a postural exercise program on VJ height in young female volleyball players with knee valgus. Methods: This pilot study included 19 young female volleyball players divided into the following groups: the Valgus Experimental Group (VEG); the Valgus Control Group (VCG); and the Neutral Control Group (NCG). All three groups carried out the same volleyball training program. In addition, only the VEG underwent a 3-month postural exercise program of 30–45 min/session, twice/week. VJ performance was measured through the Sargent test before (T0), at 6 weeks (T1), and at 12 weeks (T2). Results: A significant effect from T0 to T1 (*p* = 0.0017) and from T0 to T2 (*p* = 0.0001) was found in the VEG. No significant differences were found over time in the VCG and in the NCG. Conclusion: An integrated postural exercise program might lead to a more balanced muscle efficiency inducing athletes to obtain a higher VJ performance.

## 1. Introduction

Volleyball is a sport in which the ability to jump is a fundamental characteristic for players and the vertical jump (VJ) performance represents a key factor both in attack and defense actions [1]. In fact, being able to jump as high as possible is determinant during a block to counter the opponent’s attack, during a serve to give the optimal direction and angle, and during a spike to hit the ball, at any height, in the best possible way [2].

During a match, the number and the type of jumps varies based on the position in the pitch in both male and female players [1,3,4]. As for female players, in a study by Tillman et al. (2004), authors quantified the number of jumps performed in elite female volleyball players belonging to four teams during two matches finding an average of nearly 22 jumps per player during a game [3]. Another study in young elite volleyball players examined player jump frequency during training and matches, detected in female players an average of 41.9 jumps/h in five matches (corresponding to a total of 7.7 h of playing and 21 sets), and regarding jump frequency according to player position, authors found the lowest number of jumps per set for the libero (0.2) and the highest jump frequency for the diagonal (14.7) [5].

Besides the importance for sport success, VJs are related to the increase in injury risk, particularly for knees [5,6]. As a matter of fact, patellar tendinopathy, also called jumper’s knee, is the most common overuse injury in volleyball players, and it is strictly related to the number and height of the jumps [5,7,8]. 

Moreover, it is widely recognized that the knee valgus position is related to several knee injuries such as to the patellofemoral joint [9] and to the anterior cruciate ligament (ACL) [10] due to the relevant contribution of this joint both in the take-off and landing phases of the jump [11,12]. It is worth noting that young female athletes who participate in jumping sports, such as volleyball, have a 4–6 times higher ACL injury rate than males [10]. Moreover, the inadequate neuromuscular control during dynamic movements is considered the main risk factor for ACL injury, with a subsequent motion asymmetry and inept movement strategy. Therefore, effective training to reduce these injuries is mandatory in sports involving rapid stopping, cutting, and changing of direction (i.e., soccer, basketball, and volleyball) [13]. In fact, about 70–80% of ACL injuries are the consequences of non-contact mechanism associated with landing from a jump, changing of direction, or sudden deceleration [14].

Notwithstanding a knee valgus position is related to the increase in injury risk and even though it has also been shown to affect negatively VJ performance in both genders, with females performing worse than males [15,16], there are a lack of studies on the relationship between knee valgus and performance.

Hence, although monitoring the characteristics of the external load of the jumps is essential for evaluating the effectiveness of the training program on VJ height, several aspects such as physical characteristics of the players should be considered [1,17,18].

Based on these premises, designing training programs including integrative exercises that take into account the morphology of the players’ knees could have a beneficial effect not only on the prevention of knee injuries but also on the VJ performance.

Thus, the aim of this study was to investigate the effects of a postural exercise program, integrative to the training program, on VJ height in young female volleyball players with knee valgus. We hypothesized an improvement on VJ performance in athletes with knee valgus who performed the postural exercise program.

## 2. Materials and Methods

### 2.1. Study Design and Participants

In this pilot study participants, to be included, had to be female volleyball players, under the age of 18, with neutral knee or knee valgus. The diagnosis of knee valgus had to be in the possession of the participants and previously carried out by a specialist medical doctor. Participants were excluded if they had had injuries in the 6 months prior to enrollment in the study.

Participants included in the study were young female volleyball players with neutral knee (NK) or with knee valgus (KV). 

The entire sample was divided into the following groups: the Neutral Control Group (NCG), the Valgus Experimental Group (VEG), and the Valgus Control Group (VCG). The NCG was composed of athletes with NK while both the VEG and the VCG were composed of athletes with KV. Participants with KV were randomly allocated to the VEG or the VCG by a single investigator (D.Z.). 

All three groups carried out the same volleyball training program. In addition, only the VEG underwent a 3-month postural exercise program of 30–45 min/session, twice/week.

The measurement of VJ height was performed before (T0), at 6 weeks (T1), and at 12 weeks (T2) of the 3-month postural exercise program by a researcher who was blinded to the allocation of the participants (V.G.). 

Parents of the participants provided an informed written consent to participate in the study which, conducted in accordance with the recommendations of the Declaration of Helsinki, was approved by the Ethics Committee of the Faculty of Sport and Physical Education of the University of Novi Sad (No. 44-02-02/2018-1).

### 2.2. Postural Exercise Program

All three groups carried out the same volleyball training program. In addition, only the VEG carried out the postural exercise program.

The postural exercise program lasted 3 months with a frequency of two sessions per week lasting 30–45 min each. Specifically, as detailed in Table 1, the postural exercise program consisted of (1) strength exercises; (2) stretching exercises; and (3) proprioception exercises. In the first month, the sessions lasted 30 min and included 6 exercises, in the second month the sessions lasted 35 min and included 7 exercises, in the third month the sessions lasted 45 min and included 8 exercises. The 6 exercises carried out in the first month were also performed in the second month and in the third month in which 1 exercise and 2 exercises were added, respectively.

### 2.3. Vertical Jump Height Assessment

VJ performance of each participant was measured through the Sargent test before (T0), at 6 weeks (T1), and at 12 weeks (T2) of the 3-month postural exercise program.

The assessment required that each participant, positioned sideways to the wall on which a meter was posted, touched the wall at the highest possible point with the fingers of the hand (height 1).

Then, each participant was asked to perform a VJ and to touch the wall at the highest point reachable with the fingers of the hand (height 2).

VJ height was calculated as the difference between the two points (height 2—height 1) measured in cm.

Each participant performed 3 VJ trials with 60 s of recovery between them and the best of the 3 trials was considered for statistical analysis.

### 2.4. Statistical Analysis

Descriptive statistics, using means and standard deviations, were performed to present data. Post hoc power analysis using G*Power software 3.1.9.2 (Heinrich Heine University, Düsseldorf, Germany) was computed to estimate the level of statistical power achieved for the sample size involved. Shapiro–Wilk test was carried out to examine the normality of data distribution. 

A general linear model repeated measures was used to determine any within–between subject difference. In detail, a mixed-factor repeated-measures ANOVA was calculated to compare VJ performances over time (i.e., T0, T1, T2) and across groups (i.e., VEG, VCG, NCG). The within-subjects factor was the time, and the between-subjects factor was the group. This analysis was carried out to examine the interaction effect (i.e., time × group), and the factors main effects (i.e., time main effect, and group main effect) on the dependent variable (VJ height). In presence of significant time × group interaction effect, significant time main effect, or significant group main effect, further analyses were conducted. In detail, a one-way repeated-measures ANOVA with Bonferroni post hoc test for between-subject multiple comparisons, or with Dunnett’s post hoc test for within-subject multiple comparisons using T0 as control time, was carried out.

Partial eta-squared was used to assess the effect size. For all statistical analyses, the significance level was at *p* < 0.05. 

Data were analyzed using IBM SPSS software 23.0 (IBM Corporation, Armonk, New York, NY, USA) and GraphPad Prism 7 (GraphPad Software Inc., San Diego, CA, USA).

Boxplots and graphs were created using GraphPad Prism 7 (GraphPad Software Inc., San Diego, CA, USA).

## 3. Results

A sample of 19 young female volleyball players (10.5 ± 0.5 years; 138.4 ± 5 cm; 36.7 ± 2.2 kg) were included in the study. Post hoc power analysis showed that with a total sample size of 19 participants, we achieved a power of 62%. Shapiro–Wilk test showed that participants with NK (*n* = 8) and with KV (*n* = 11) were normally distributed. After the random allocation of the participants with KV, the entire sample was divided into the following groups: the Neutral Control Group (NCG; *n* = 8; 10.6 ± 0.5 years; 140.9 ± 5.9 cm; 36.03 ± 2.3 kg); the Valgus Experimental Group (VEG; *n* = 8; 10.6 ± 0.5 years; 137.8 ± 3 cm; 37.5 ± 2.1 kg); and the Valgus Control Group (VCG; *n* = 3; 10.7 ± 0.03 years; 133.4 ± 2.4 cm; 35.9 ± 1.8 kg).

**Figure 1:**
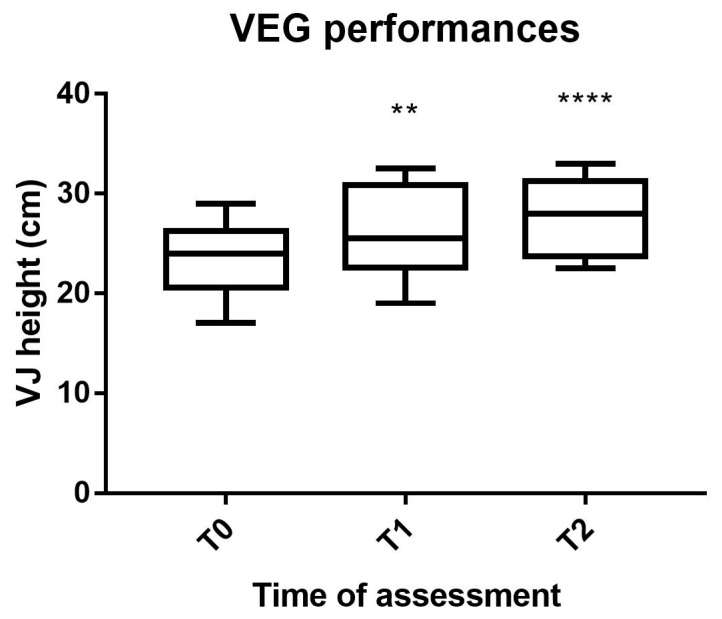
VEG performances. Legend: VEG, Valgus Experimental Group; VJ, vertical jump; T0, pre-intervention assessment; T1, mid-intervention assessment; T2, post-intervention assessment; **, *p* < 0.01; ****, *p* < 0.0001.

**Figure 2:**
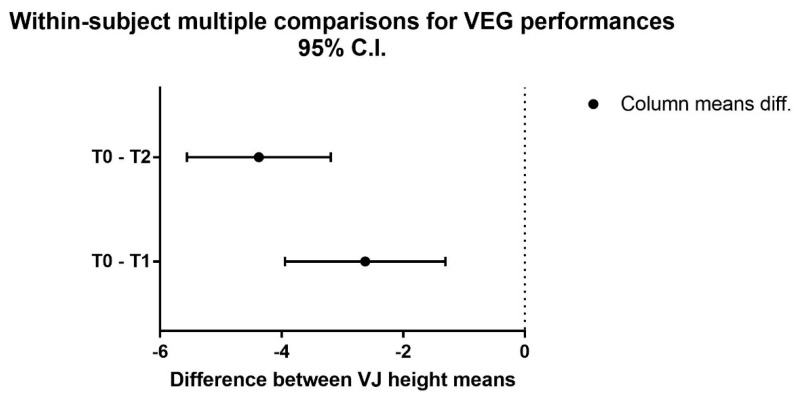
Multiple comparisons for VEG performances. Legend: VEG, Valgus Experimental Group; T0, pre-intervention assessment; T1, mid-intervention assessment; T2, post-intervention assessment.

The mixed-factor repeated-measures ANOVA detected no significant interaction time × group, although almost statistically significant (F(4,32) = 4.55, *p* = 0.050, η^2^ = 0.250) representing a trend. No significant differences across the three groups were found, while a significant difference across the three time point was detected [F(2,32) = 25.51, *p* < 0.0001, η^2^ = 0.483]. Based on the significant time main effect observed, the one-way repeated-measures ANOVA showed a significant difference over time in the VEG [F(2,14) = 41.51, *p* < 0.0001] (Figure 1) with the Dunnett’s multiple comparisons test that showed a significant effect from T0 to T1 (*p* = 0.0017) and a higher significant effect from T0 to T2 (*p* = 0.0001) (Figure 2).

The one-way repeated-measures ANOVA detected no significant differences over time in the VCG and in the NCG.

Means and standard deviations of VJ performances, and results from the one-way repeated-measures ANOVA and the Dunnett’s multiple comparisons test are reported in Table 2. 

## 4. Discussion

The aim of this study was to explore the effects of a 3-month postural exercise program on VJ height in young female volleyball players with knee valgus, hypothesizing an improvement in VJ performance after the postural exercise program.

In line with the hypothesis, our results showed that young athletes who performed the postural exercise program reached a higher VJ performance both after 6 weeks (T1) and after 12 weeks (T2) of the postural exercise program. No differences were found at T1 and at T2 in athletes with knee valgus who did not perform the postural exercise program.

Despite the extensive literature on the association between knee valgus and the risk of related injuries, primarily anterior cruciate ligament (ACL) injury and patellofemoral pain (PFP), very few studies have investigated the influence of the knee valgus on performance, and this limits the comparison of findings that we found with previous studies [10,19,20]. 

Besides this premise, our findings can be explained according to a biomechanical approach that take into account, firstly, the biomechanical contribution of hip, knee, and ankle joints on VJ performance. In fact, during the VJ the biomechanical contribution of each joint of the lower limb is almost equivalent (about 33% for both hip, knee, and ankle) both in the take-off and in the landing phase [11]. On the basis of its contribution and as reported by previous studies, since the knee valgus position during the landing phase is associated with the injury rate to the ACL, we assume that, in a similar way, the performance of VJ height could be related to the knee valgus considering that its contribution with respect to the other joints is the same as in the landing phase. This assumption is supported by Kotsifaki et al. (2021) in which authors suggested that, considering the similar contribution of each lower limb joint, any dysfunction in hip, knee, or ankle joint may negatively affect VJ height [11]. In particular, the authors emphasize the importance of the knee with respect to the hip and ankle especially in the vertical jump compared to the horizontal jump where the contribution appears to be lower [11]. A seminal work even reported that the knee produces 49% of the total positive work with the remainder distributed roughly equally by the hip and ankle [21]. In fact, in line with our findings, it has been demonstrated that a tibial-femoral misalignment during the take-off phase can cause atypical loading patterns in volleyball players, negatively affecting performance [8].

The second biomechanical aspect concerns the muscles that contribute during each VJ phase, i.e., take-off, flight, and landing. In particular, previous studies have emphasized the role of bi-articular muscles analyzing the contribution of rectus femoris, vastus, soleus, gastrocnemius, gluteus, and hamstrings on VJ performance [22,23,24]. However, a misalignment between femur and tibia, which is present in the knee valgus, can result from weakness in the quadriceps muscles, calf muscles, and hip abductors muscles [25]. Moreover, since knee valgus can be caused by a femoral anteversion and/or an external tibial torsion, these conditions increase the adductor muscles stress, negatively affecting a task such as gait pattern [25,26,27,28]. For example, the semimembranosus along with semitendinosus muscle are located in the medial side of the posterior thigh compartment and, based on their distal insertion, are responsible for the knee internal rotation as in the knee valgus [29]. As a consequence, we suggest that these conditions may cause differences in both muscle tone and strength of some muscles and our targeted integrated intervention improved muscle balance leading to reach higher VJ height. In fact, it is widely known that postural exercises, as for a knee dysfunction such as knee valgus, led to a more balanced muscle efficiency inducing athletes to obtain higher performances [30,31,32,33,34].

The limitations of our study concern the sample size, even if this depends on the recruitment of athletes with peculiar morphological characteristics of the knee and the lack of previous studies that have analyzed the relationship between knee valgus and VJ performance in athletes which limits the comparison of results. However, the originality of the topic is, at the same time, the major strength of the study.

## 5. Conclusions

In conclusion, our study suggests integrating sport-specific training with personalized exercise programs based on the morphological characteristics of young athletes in order to improve their performance.

## Figures and Tables

**Table 1:** Postural exercise program.

1st month			
Exercise 1	Proprioception exercise	Maintain the upright stance in monopodal position on a balance disc with the flexion of the contralateral hip for 30 s.	Rest period: 30 s Duration of the exercise: 5 min
Exercise 2	Proprioception exercise	- Walk for 20 m first on the tips of the feet, then on the heels of the feet, and then on the outer edge of the feet. - Walk for 20 m first on the tips of the feet, then on the heels of the feet, and then on the outer edge of the feet adding flexion, abduction, and external rotation of the hip with each step.	Rest period: 30 s Duration of the exercise: 5 min
Exercise 3	Strength exercise (Hip abductor muscles)	In standing position facing the wall with the hands resting on it and an elastic band around the knees, abduct the knees and hold them for 3 s.	Repetitions: 15 Sets: 3 Rest period: 2 min
Exercise 4	Strength exercise (Hip abductor muscles + Quadriceps muscles)	In a sitting position on a chair with the lower limbs bent and an elastic band around the knees, extend the knees and abduct the knees and hold them for 3 s.	Repetitions: 15 Sets: 3 Rest period: 2 min
Exercise 5	Stretching exercise (Hip adductor muscles)	In a sitting position on the floor with the lower limbs bent, abducted, externally rotated, and with the soles of the feet in contact with each other, gently push the knees down with the hands and hold the position for 30 s.	Repetitions: 3 Sets: 1 Rest period: 30 s
Exercise 6	Stretching exercise (Hip internal rotation muscles)	Maintain the sitting position on the floor for 30 s with: a lower limb bent, and the hip externally rotated in front of the body so the lower leg and knee are resting on the ground with the knee forming a 90-degree angle; the other lower limb beside the body with the hip internally rotated so the lower leg and ankle are resting on the ground with the knee forming a 90-degree angle.	Repetitions: 3 × leg Sets: 1 Rest period: 30 s
2nd month			
Exercise 7	Proprioception exercise	Walk for 10 m in a path in which each step is carried out on balance discs.	Rest period: 30 s Duration of the exercise: 5 min
3rd month			
Exercise 7	Strength exercise (Hip abductor muscles + Gluteus muscles + Quadriceps muscles + Hamstrings muscles)	In standing position with an elastic band around the knees, abduct the knees and hold them for 3 s and then perform a 90-degree squat maintaining the knees abducted.	Repetitions: 15 Sets: 3 Rest period: 2 min
Exercise 8	Stretching exercise (Hamstrings muscles + Hip adductor muscles + Hip internal rotation muscles)	Maintain the sitting position for 5 min with the back on the floor and the lower limbs extended on the wall; then with the hip externally rotated; then with the hip abducted.	Duration of the exercise: 5 min

Legend. 2nd month = 1st month exercises + exercise 7; 3rd month = 1st month exercises + exercises 7 and 8.

**Table 2:** VJ performances before, at 6 weeks, and at 12 weeks.

Group	T0	T1	T2		T0 vs. T1		T0 vs. T2	
	(cm)	(cm)	(cm)	Lev. Sig.	Mean Diff.	Lev. Sig.	Mean Diff.	Lev. Sig.
VEG	23.31 ± 3.79	25.94 ± 4.65	27.69 ± 3.79	*p* < 0.0001	−2.625	*p* = 0.0017	−4.375	*p* = 0.0001
VCG	26.67 ± 4.16	27.67 ± 3.69	28.00 ± 3.46	n.s.	−1.00	-	−1.333	-
NCG	29.88 ± 6.31	30.38 ± 5.51	31.88 ± 5.57	n.s.	−0.5	-	−2.00	-

Legend. T0, pre-intervention assessment; T1, mid-intervention assessment; T2, post-intervention assessment; VEG, Valgus Experimental Group; VCG, Valgus Control Group; NCG, Neutral Control Group.

## Data Availability

The data presented in this study are available on request from the corresponding author.
